# A Bifunctional Molecule with Lectin and Protease Inhibitor Activities Isolated from *Crataeva tapia* Bark Significantly Affects Cocultures of Mesenchymal Stem Cells and Glioblastoma Cells

**DOI:** 10.3390/molecules24112109

**Published:** 2019-06-04

**Authors:** Camila Ramalho Bonturi, Mariana Cristina Cabral Silva, Helena Motaln, Bruno Ramos Salu, Rodrigo da Silva Ferreira, Fabricio Pereira Batista, Maria Tereza dos Santos Correia, Patrícia Maria Guedes Paiva, Tamara Lah Turnšek, Maria Luiza Vilela Oliva

**Affiliations:** 1 Department of Biochemistry, Federal University of São Paulo, 04044-020 São Paulo, SP, Brazil; camilabntr@gmail.com (C.R.B.); mariana.cabral@kroton.com.br (M.C.C.S.); bruno_salu@hotmail.com (B.R.S.); rodrigobioq@gmail.com (R.d.S.F.); fabriciopbat@gmail.com (F.P.B.); 2 Department of Biotechnology, Jozef Stefan Institute, 1000 Ljubljana, Slovenia; helena.motaln@ijs.si; 3 Department of Biochemistry, Federal University of Pernambuco, 50670-910 Recife, PE, Brazil; mtscorreia@gmail.com (M.T.d.S.C.); ppaiva63@yahoo.com.br (P.M.G.P.); 4 Department of Genetic Toxicology and Cancer Biology, National Institute of Biology, 1000 Ljubljana, Slovenia

**Keywords:** CrataBL, glioblastoma, mesenchymal stem cells, microenvironment, plant lectin, protease inhibitor

## Abstract

Currently available drugs for treatment of glioblastoma, the most aggressive brain tumor, remain inefficient, thus a plethora of natural compounds have already been shown to have antimalignant effects. However, these have not been tested for their impact on tumor cells in their microenvironment-simulated cell models, e.g., mesenchymal stem cells in coculture with glioblastoma cell U87 (GB). Mesenchymal stem cells (MSC) chemotactically infiltrate the glioblastoma microenvironment. Our previous studies have shown that bone-marrow derived MSCs impair U87 growth and invasion via paracrine and cell–cell contact-mediated cross-talk. Here, we report on a plant-derived protein, obtained from *Crataeva tapia* tree Bark Lectin (CrataBL), having protease inhibitory/lectin activities, and demonstrate its effects on glioblastoma cells U87 alone and their cocultures with MSCs. CrataBL inhibited U87 cell invasion and adhesion. Using a simplified model of the stromal microenvironment, i.e., GB/MSC direct cocultures, we demonstrated that CrataBL, when added in increased concentrations, caused cell cycle arrest and decreased cocultured cells’ viability and proliferation, but not invasion. The cocultured cells’ phenotypes were affected by CrataBL via a variety of secreted immunomodulatory cytokines, i.e., G-CSF, GM-CSF, IL-6, IL-8, and VEGF. We hypothesize that CrataBL plays a role by boosting the modulatory effects of MSCs on these glioblastoma cell lines and thus the effects of this and other natural lectins and/or inhibitors would certainly be different in the tumor microenvironment compared to tumor cells alone. We have provided clear evidence that it makes much more sense testing these potential therapeutic adjuvants in cocultures, mimicking heterogeneous tumor–stroma interactions with cancer cells in vivo. As such, CrataBL is suggested as a new candidate to approach adjuvant treatment of this deadly tumor.

## 1. Introduction

In nature, plant lectins are essential for their viral, bacterial, and fungal defense properties. Legume lectins emerge in human diet, being present in foods like tomato, peanut, banana, lentil, soybean, rice, potato, and others. When consumed, lectins’ biological activity may be preserved, as they are resistant to gut digestion, enabling their possible function, even in the circulation system [1]. Plants lectins are used as potential biomarkers for several diseases as they are associated with antimicrobial, insecticidal, and antitumor activity [1,2,3]. As lectins bind to carbohydrates moieties, they detect large and heterogeneous group of proteins in tumor cancer tissues, since glycosylated proteins are commonly upregulated in cancer, where their various functions tend to increase tumor progression and other aberrant conditions [4,5,6]. CrataBL is a natural compound isolated from *Crataeva tapia* tree. The “BL” in CrataBL stands for bark lectin origin, as the protein was extracted from *Capparaceae* bark grown in northeastern Brazil [7]. It has a dual function, as besides its lectin activity, it acts as a 20 kDa Kunitz-type serine protease inhibitor, inhibiting trypsin (43 µM) and human factor Xa (8.6 µM) [8]. CrataBL lectin capacity was demonstrated by its specificity to bind sulfated oligosaccharides [7,8,9]. This protein affects the development of larvae of *Callosobruchus maculatus* [2], prolongation of blood coagulation, reduction of occlusion time of arterial flow [9], and glycemia in diabetic mice [10]. In a cancer model, it has been reported that CrataBL induced apoptosis of prostate cancer cell lines DU-145 and PC-3 [7].

Glioblastoma (GB) are classified as rare cancers, yet they the most common and aggressive among brain cancers with still no efficient treatment, reflected in their early recurrence. Tumor cell-autonomous heterogeneity [11] and treatment-dependent plasticity of recurrent vs. primary glioma subtypes hamper efficient radiation and chemotherapy [11,12]. Although several synthetics and natural compounds have been proposed as adjuvant therapeutics to standard radiotherapy, no effective breakthrough in treatment of GB has yet been achieved. On the other hand, tumor cell-nonautonomous heterogeneity of GB, comprising a plethora of stromal cells infiltrating the tumor, presents the obstacle to successful treatment. Thus, novel strategies, including ones involving mimetics of the GB microenvironment, should be considered [12,13].

Human mesenchymal stem cells (MSCs) are adult, nonhematopoietic, multipotent progenitor cells, originally isolated from the bone marrow, which are traditionally characterized in vitro by their plastic adherence, trimesenchymal differentiation, and expression of a panel of distinguishing surface markers [14]. The interaction of human mesenchymal stem cells (hMSCs) and tumor cells has been investigated in various contexts. MSCs are considered as cellular treatment vectors based on their capacity to migrate towards a malignant lesion. However, concerns about the unpredictable behavior of transplanted MSCs are accumulating markers [13]. Mesenchymal stem cells are part of GB stromal components and have also been investigated for cellular GB treatment due to their ability to modulate glioblastoma cell phenotype [15,16]. However, the mechanisms by which MSCs affect various types of cancers remain controversial and include mediation by cytokines, their receptors, and growth factors [15,16,17,18,19,20]. Of these, priming of toll-like receptors TLR3 and TLR4 seems to significantly affect MSC interactions with tumor cells and we propose this to be the key role in MSC and GB cross-talk via CCL2/MCP1 cytokines [21,22]. On the other hand, MSCs may be capable of delivering therapeutics to the brain, as they can transverse the blood–brain barrier under pathological conditions [17,20,21,22]. Considering that, we aimed to characterize CrataBL’s effects on glioblastoma and its microenvironment, mimicked here by direct coculturing of GB cells with MSCs.

## 2. Results

### 2.1. CrataBL Impaired Cell Viability and Induced Cell Death

**Figure 1:**
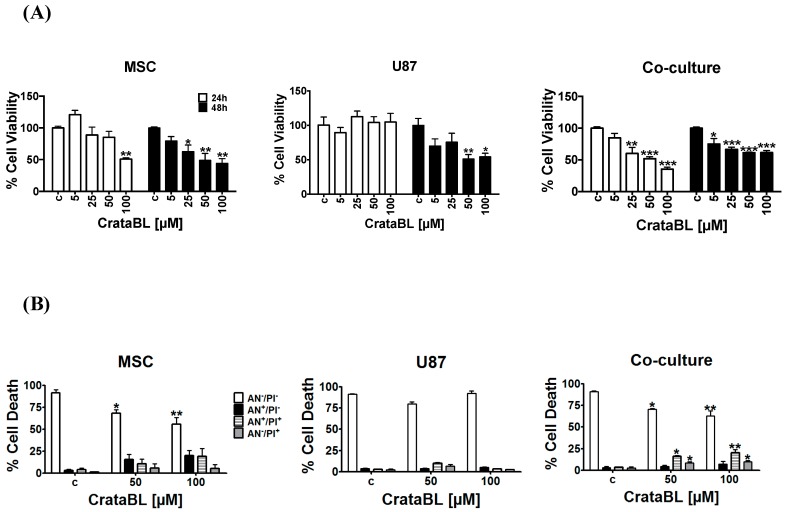
Effects of CrataBL on the cell viability and cells death of MSCs, U87 cells, and cocultured cells. Cell viability was measured by MTT assay at increasing concentrations of the inhibitor CrataBL at 5 µM, 25 µM, 50 µM, and 100 μM concentrations after 24 and 48 h of treatment. The absorbance values were normalized to nontreated control cells (c), as described in the Methods section. Panels represent (**A**) MSC, U87 glioblastoma cells, and direct cocultures. Cell death (**B**) was evaluated by flow cytometry as described in the Methods section, in MSC, U87 cells and cocultures after 24 h of CrataBL treatment. AN^−^/PI^−^ represent viable cells; AN^+^/PI^−^ represent early apoptotic; AN^+^/PI^+^ and AN^−^/PI^+^ represent late apoptotic and necrotic cells. Significance among experimental groups was considered as * *p* < 0.05, ** *p* < 0.005, and *** *p* < 0.0005.

CrataBL had stronger effects on the viability of MSC than on the U87 cells. Whereas the viability of MSC was affected after 24 h treatment only at the 100 µM dose, the viability of U87 cells remained unchanged; however, CrataBL significantly impaired the viability of both cell lines after 48 h treatment. On the contrary, treatment with CrataBL reduced the viability of cells in cocultures after 24 h in a dose-dependent manner (Figure 1A). This indicates that MSCs enhance the effect of CrataBL in decreasing the viability of U87 cells in coculture. To verify whether the reduction of cell viability resulted from the induction of cell death, flow cytometry coupled to annexin V (AN) and propidium iodide (PI) staining was used to distinguish the live (AN^−^/PI^−^), early apoptotic (AN^+^/PI^−^), late apoptotic (AN^+^/PI^+^), and necrotic (AN^−^/PI^+^) cells in cultures exposed to CrataBL for 24 h. Although the viability of MSCs was affected by CrataBL, no significance in the number of early, late, or necrotic cells was detected after 24 h treatment. Consistent with the viability data, no decrease in the apoptotic or necrotic cells counts was detected in treated U87 cells, suggesting that CrataBL alone does not induce cell death pathways in these cells. In contrast, in cocultures treated with 50 µM and 100 µM of CrataBL, an increase in late apoptotic and necrotic cells was detected (Figure 1B). The fact that the mixed cocultures were more sensitive to the inhibitor than each of the individual cell types may suggest that their interactions would render the heterogeneous tumor more refractory to CrataBL.

### 2.2. CrataBL Affected Proliferation and Cell Cycle

**Figure 2:**
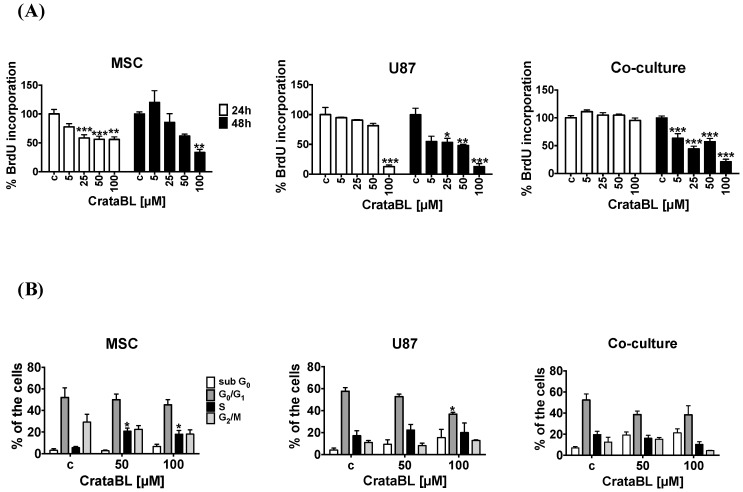
Effects of CrataBL on the proliferation of MSCs, U87 cells, and cocultured cells. Proliferation was determined by cell cycle assay using flow cytometry. Panels represent (**A**) MSC, U87 glioblastoma cells, and direct cocultured cells, in the presence of increasing concentrations of CrataBL inhibitor as measured by BrdU assay, relative (%) to nontreated control cells (c). Cell cycle phase distribution was determined by propidium iodide staining followed by flow cytometry analyzes (**B**). Panels represent MSC, U87 GB cells, and cocultured cells, treated with CrataBL (50 µM and 100 µM) for 24 h and then permeabilized. Percentages of cells in sub G_0_, G_0_/G_1_, S, and G_2_/M phase were determined using Accuri C6 cytometer, with at least 10,000 events collected. Significance was considered at * *p* < 0.05, ** *p* < 0.005, and *** *p* < 0.0005.

The proliferation, measured by BrdU incorporation, was affected already after 24 h for nearly 40% and only in the high dose after 48 h. In contrast, CrataBL inhibited proliferation of U87 cells already at lower doses after 48 h and in the high dose after 24 h. In the cocultures, CrataBL treatment strongly decreased their proliferation upon 48 h (Figure 2A) in a similar pattern as observed with U87 cells. Flow cytometry analysis was used to monitor the percentage of cells in each cell cycle phases (G_1_, S, G_2_, or M phase) in response to CrataBL treatment. The percentage of treated MSCs residing in the S phase of the cell cycle was increased, and in U87, the percentage of the cells in G_0_/G_1_ phase was decreased. No alterations in the cell cycle in the cocultures (Figure 2B) were observed. Still, the percentage of cells residing in the resting, sub-G_0_ phase showed tend to increase in U87 cell monoculture and in coculture conditions upon CrataBL treatment, possibly meaning that the cells were stopped at G_0_/G_1_ checkpoints, but CrataBL had a minor, non significant effect on cocultured cells.

### 2.3. CrataBL Reduced Invasion and Metalloprotease Activity

**Figure 3:**
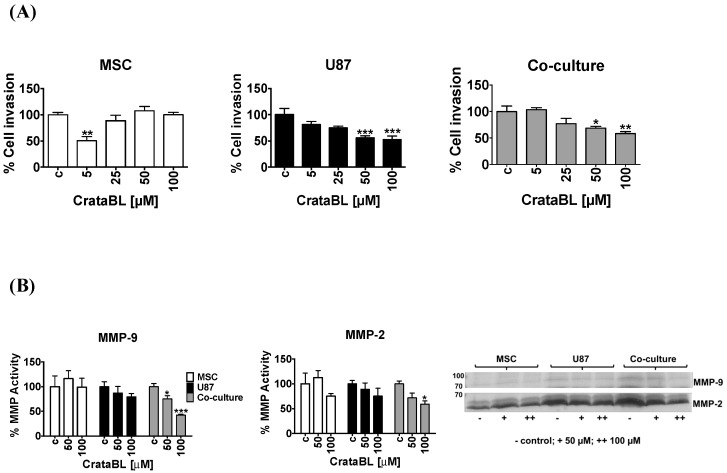
The effects of CrataBL on the invasion and metalloproteases activity secretion of MSCs, U87 cells, and cocultured cells. The invasion through the matrigel layer was evaluated as the percentage of invasion normalized to nontreated cells as control (c) in MSC, U87 glioblastoma cells, and cocultured cells (**A**). CrataBL influenced metalloprotease activity as observed by gelatin zymography gel assay and quantified for metalloproteases MMP-9 ±84/92 kDa and MMP-2 ±64/72 kDa (**B**). Densitometry analyses showed a diminished activity of MMP-2 and MMP-9 in the cocultures. (−) indicates control, (+) indicates 50 µM of CrataBL and (++) 100 µM of CrataBL in MSC, U87 and coculture. Significance was considered at * *p* < 0.05, ** *p* < 0.005, and *** *p* < 0.0005.

Cancer cell invasion is characterized by degradation of the surrounding extracellular matrix and their spread into the adjacent tissues. While MSC appeared not to be affected by the 50 µM and 100 µM inhibitor treatment after 24 h, U87 cells at the same CrataBL concentrations were significantly less invasive. In cocultures, the inhibitory activity followed the U87 cells’ pattern (Figure 3A), pointing to the fact that CrataBL selectively inhibited invasion of cancer cells in the cocultures.

Next, we evaluated the CrataBL treatment on MMP-9 and MMP-2 since these gelatinolytic MMPs play important a role in invasion by degrading native extracellular matrix protein components or otherwise affecting protease signaling [23]. As shown in Figure 3B, the gelatinolytic activity of MMPs appears as a translucent band in the images, and the band intensity was analyzed by densitometry, as described in the Methods section. The molecular weight of MMP-9 was predicted as ±84/92 kDa and MMP-2 ±64/72 kDa. Using a metalloprotease activity blocking buffer, no bands were observed, suggesting that the observed gelatinolytic activities corresponded to MMPs (data not shown). As demonstrated in Figure 3B, CrataBL treatment only inhibited MMP-9 and MMP-2 activity in coculture media, which parallels the decreased invasion in cocultured cells. No CrataBL effect was observed on MMP-9 and MMP-2 activity of MSCs and only a trend of invasion decrease was observed in U87 cells. These data support the notion that MMPs’ activity is only inhibited by CrataBL upon MSC interaction with U87 cells in coculture.

### 2.4. CrataBL Reduced Cell Adhesion

**Figure 4:**
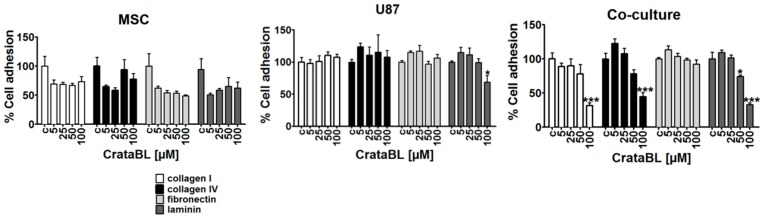
The effects of CrataBL on cell adhesion. Cell adhesion to collagen I, collagen IV, laminin, and fibronectin coatings was evaluated, as described in Methods on MSC, U87 glioblastoma cells, and cocultured cells at 5 µM, 25 µM, 50 µM, and 100 µM CrataBL concentrations, normalized to nontreated cells as control (c). Significance was considered at * *p* < 0.05, and *** *p* < 0.0005.

During tumor progression, the synthesis and composition of extracellular matrix (ECM) components by cancer cells are altered and are partially degraded, e.g., an increase of collagen fibers, culminating in cancer invasion and metastatic spread. Such alterations in the extracellular matrix network, consisting of collagen I and IV, fibronectin and in brain tumors, in particular, the most abundant ECM component laminin, may contribute to altered molecular mechanisms of invasion [24]. In MSCs, no significant alterations in cell adhesion by CrataBL treatment were observed, regardless of the substrate used. As expected, U87 cells’ adhesion decreased on laminin, the native brain substrate for glioblastoma cells upon treatment with CrataBL. Accordingly, in coculture conditions, impaired cell adhesion on collagen I and IV, as well as in laminin, but not fibronectin, was observed when cells were treated with higher concentrations (50 and 100 µM) of CrataBL (Figure 4).

### 2.5. CrataBL Altered Cytokine Levels and Nitric Oxide Release

**Figure 5:**
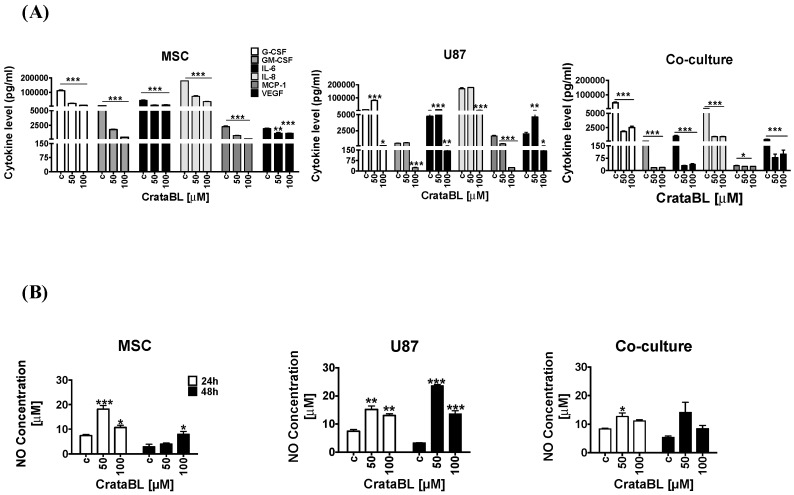
Secreted cytokines profiling and nitric oxide release. Cytokines (G-CSF, GM-CSF, IL-6, IL-8, MCP-1, and VEGF) release following CrataBL treatment with 50 µM and 100 µM after 24 h in the media of (**A**) MSC, U87 glioblastoma cells, and cocultured cells. Nitric oxide release was analyzed by chemiluminescence in (**B**) MSC, U87 glioblastoma cells, and cocultured cells after 24 h and 48 h of CrataBL treatment (50 µM and 100 µM), compared to nontreated controls (c). Significance was considered at * *p* < 0.05, ** *p* < 0.005, and *** *p* < 0.0005.

To verify whether the CrataBL inhibitory effects are possibly mediated by the inflammatory cytokines and chemokines playing a role in GB progression [25], the selected chemokines’, i.e., G-CSF, GM-CSF, IL-6, IL-8, MCP-1, and VEGF, as well as nitric oxide (NO), release was analyzed in mono and cocultured media upon CrataBL treatment. This inhibitor decreased the levels of all the above cytokines/chemokines in MSC, U87 cells, and cocultures (Figure 5A). The decrease was more abrupt in cocultures when compared to monocultured cells. These results may be linked to the increase in nitric oxide (NO) production upon addition of CrataBL after 24 h and 48 h in MSC and in U87 cell monocultures. In U87 cell culture, a nearly four-fold increase in NO release was detected under 50 μM CrataBL treatments after 48 h. In coculture, the changes in NO release were less prominent, due to yet unknown interactions between MSC and U87 cells (Figure 5B).

## 3. Discussion

Although much attention is given to cell autonomous mechanisms of tumor progression [11], non-cell-autonomous mechanisms, particularly interactions between tumor cells and stromal cells, are increasingly recognized as important contributors to tumor growth and resistance to therapy [12]. Compared with other cancers, the stroma of GB is not well understood, due to the uniqueness of the brain and it is thought to be composed of reactive astrocytes, endothelial cells, and immune cells. However, the contributions of other cell types, such as MSCs, have not been studied to a great extent. MSCs are also known for their ability to migrate to zones of tissue injury and tumors, being attracted to inflammatory cytokines which are produced in the cancer microenvironment. We have previously demonstrated that glioblastoma cells, as well as MSCs, differentially express connexins and that they interact via gap-junctional coupling. Besides this so-called functional syncytium formation, Schichor et al. [26] have also provided evidence of cell fusion events (structural syncytium). Direct and indirect interactions between mesenchymal stem cells (MSCs) and cancer cells in vitro and in vivo have been shown to either negatively or positively affect the malignant phenotype of cancer cells [27,28,29], even inducing their senescence and dormancy [27] as well as cell fusion in direct cultures, forming cancerous hybrid cells and exhibiting entosis (cell cannibalism), as observed recently by Oliveira et al. [18].

We have demonstrated that CrataBL diminished the viability of MSCs to a greater extent than in U87 cells, this being in agreement with previous reports by Ferreira et al. [8] in prostate cancer. However, the inhibition of mixed cells grown in 1:1 coculture, mimicking glioblastoma in vivo, where MSCs would infiltrate the tumor was even more affected by CrataBL than U87 cells alone. This indicates that there is cellular cross-talk between these two cell types, rendering the coculture less viable (Figure 1).

Furthermore, we investigated the processes that may affect cell viability, such as cell death being induced in the cocultures of U87/MSC. An increase in the percentage of late apoptotic and necrotic cells was observed in U87/MSC cocultures upon 24 h of CrataBL treatment (Figure 1A). We believe that at this time point, MSCs in monocultures and in the cocultures were affected first. This lowers total coculture cell viability in 24 h. However, the total cell proliferation (division, Figure 2A), due to more efficiently dividing U87 cells, in this coculture compensated for the lowered proliferation of MSCs after 48 h. This corroborates previous observations in U87/MSC [18]. It is noteworthy that BrdU incorporation does not discriminate the types of cells in coculture, and U87 stopped dividing only after a prolonged time, both alone and in the coculture (48 h), demonstrating that these cells are more resistant to CrataBL. The decrease of total cell viability, as shown in Figure 1A, in cocultured cells at 24 h is thus due to dying MSCs, as these cells were more vulnerable to the apoptotic effect of the inhibitor, as one would expect (Figure 1B). Interestingly, these results are in contrast to our previous experiments with another protease inhibitor (EcTI), isolated from *Enterolobium contortisiliquum*, also of the same Kunitz-type family, but having no lectin-binding properties [30]. EcTI did not cause U87 cell death, neither in mono, nor in coculture, suggesting that the proteolysis inhibition per se does not play a role in apoptosis. However, CrataBL increased the numbers of cells in the G_0_/G_1_ checkpoint after 24 h treatment, in U87 monocultured cells, and in mixed cocultured cells, indicating that senescence is linked to cell cycle checkpoint G_0_ phase arrest.

As CrataBL impaired metabolic activity, cancer cell cycle progression, and proliferation of the coculture, we were interested whether the remaining viable U87 cells’ invasiveness was affected by CrataBL. In the invasion experiments, we did not discriminate between U87 and MSCs that were collectively determined before and after treatment with CrataBL for 15 min. As the inhibitor did not significantly affect MSCs alone, but did affect U87 cells above 50 μM (Figure 3A), we may speculate that even if the invaded cells contained both U87 cells and MSCs, CrataBL would first bind to and inhibit the more invasive U87 cells, as they also express higher levels of metalloproteases (Figure 3B). These cells also express other proteolytic enzymes, as reported by Breznik et al. [16]. In that study, we used a differential dye and gene labeling of the MSCs and U87 cells, respectively, in 2D and in 3D cocultures. We have shown that MSCs in coculture did impair U87 invasion, but enhanced the invasion of U373 under the same coculture conditions (1:1 ratio of cells), which was associated with an increase in other proteolytic enzymes, cathepsin B and urokinase, besides MMPs. In another study, Oliveira et al. [18] used the same coculture system to expose the coculture to the inflammatory peptide bradykinin. After that, the invasion of U87 cells (but not MSCs) out of the cocultures was increased and, furthermore, after separation of U87 cells from the three-day cocultures, we found that this was due to increased expression of EMT-related genes’ induction in the glioblastoma cells.

Here, we also found that the activities of MMP-2 and MMP-9 are involved in glioblastoma cells’ invasion [31], as the activities of MMP-2 and MMP-9 were impaired in CrataBL-treated cocultures, but remained unchanged in CrataBL-treated MSC and U87 cell monocultures. This confirms that MMPs may enhance MSCs and U87 cells, as observed in our previous studies [16].

The adherence of cancer cells to extracellular matrix proteins, which in the brain is comprised mostly of laminin, and to a lesser extent of fibronectin and collagen type IV, which is needed for their subsequent degradation by GB cells, enables them to invade adjacent tissues. Here, we found that in U87/MSC cocultures, CrataBL treatment significantly inhibited U87 and U87/MSC cocultured cells’ invasion, but did not affect monocultured MSCs’ migration. Impaired invasion of U87 cells in indirect cocultures with bone marrow MSCs has previously been reported, however, in the experiments by Breznik et al. [16] and based on transcriptomes alteration of these two cell lines upon paracrine interactions. We conclude that not only MMP induction, but also enhanced binding to some of the induced adhesion molecules, such as ephrin, may play a role in cell adhesion and repulsion processes, adding to the generally reduced invasion in indirect cocultured U87-MG cells [32]. This is in contrast to the observed effects of CrataBL potentiating the MMP-induced inhibition of U87 cell adhesion to matrix proteins. Several studies have already highlighted the importance of these matrix proteins in brain tumor malignancy, angiogenesis, proliferation, and patient survival [23]. In this study, the adhesion of MSC and U87 cells alone to three ECM-related substrates remained unaltered upon CrataBL treatment, but was inhibited in U87 coculture pretreatment, especially with laminin, where the adhesion was significantly decreased at a higher (100 μM) CrataBL concentrations. CrataBL more effectively decreased U87 cell adhesion compared to the other Kunitz-type inhibitor, EcTI, which only slightly decreased the adhesion of U87 cells to fibronectin and MSC cocultured U87 cells to collagen IV [30]. CrataBL seems to interact in a more complex manner with cells to impair their adhesion to extracellular matrix components than EcTI. In parallel to adhesion, both matrix proteases were more inhibited by CrataBL compared to EcTI-treated cocultures.

Inflammatory cytokines play important roles in cancer progression, and selected cytokine therapies have already been used [32,33]. Due to accumulating evidence on the immunomodulatory effects of activated MSCs, the enhanced secretion of chemokine and cytokines when in contact with cancer cells has been observed. In indirect cocultures, we found CCL2/MCP to be the most significantly regulated chemokine in U87/MSC paracrine signaling, in addition to several other chemokines [32,34,35] that may account for changed cocultured cells’ phenotypes by affecting several genes associated with proliferation (Pmepa-1, NF-κB, IL-6, IL-1b), invasion (EphB2, Sod2, Pcdh18, Col7A1, Gja1, Mmp1/2), and senescence (Kiaa1199, SerpinB2). These have generally been found as either inhibitory or promoting cancer progression, due to several reasons, as reviewed in Lee and Hong [36]. Here, we found that in response to CrataBL treatment, the levels of selected chemokines and selected inflammatory cytokines’, including IL-6, IL-8, GM-CSF, VEGF, MCP-1, and G-CSF, secretion were effectively reduced in the mono- and cocultures, demonstrating the antitumorigenic effect of CrataBL. The cytokines IL-6 and IL-8 are both known to enhance the invasiveness of GB cells and increase angiogenesis, whereas GM-CSF is involved in proliferation and growth, and VEGF promotes angiogenesis by HIF-1alpha induction in GB. Likewise, MCP-1 (CCL-2, CC-chemokine ligand 2) facilitates GB progression, as a monocyte-attracting chemokine. GM-CSF enables glioma cells to proliferate and invade adjacent tissue [33].

Similarly, nitric oxide (NO), a multifaceted small molecule associated with the anti or procancer activity, is known to mediate the attraction of immune cells to the wound site and to act as an antiproliferative agent in cancer cells. NO may be produced constitutively or is induced after inflammatory conditions, vascular injury and/or cell proliferation [37,38]. In GB, NO release was related to the sensitivity of cancer cells to temozolomide therapy [38]. Nitric oxide release was enhanced in U87 cells as well as in MSCs treated with CrataBL. In cocultures, however, a significant increase in NO release after CrataBL treatment was diminished compared to MSCs of U86 alone. Altogether, these results confirm that the antiproliferative effect of CrataBL on U87 cells and GB/MSC cocultures may also be mediated via NO release. In GB, the MSC treatment was effective in animal experiments [39,40]. The fact that the mixed cocultures were more sensitive to the inhibitor than each of the individual cell types with respect to cell processes suggests that their interactions would render the heterogeneous tumor mass more resistant to CrataBL. It has also been demonstrated in vivo (animal and clinical studies) that heterogeneous tumors are less sensitive to treatment, due to stromal cells supporting tumor resistance.

Taken together, our research was focused whether CrataBL acts on cocultures that resemble the in vivo GB tumor microenvironment more (or less) efficiently than on monocultures of MSCs and U87 cells. We have shown that, indeed, CrataBL decreased cell proliferation, enhanced the transition from early to late apoptosis, and slightly enhanced cell proliferation in the cocultures vs. monocultures. Further, it inhibited the adhesion of mixed cells, but did not significantly inhibit the invasion out of the cocultures. CrataBL significantly affected NO release from cocultures after longer exposure, which would have an effect on cytokine release and immune response, which is undoubtedly different in a heterogeneous tumor environment compared to isolated tumor cells. Thus, we emphasize that it makes much more sense to test inhibitors or other natural compounds in cocultures that mimic heterogonous tumors, than in isolated cancer cells, as is usually done. Finally, we present the potential beneficial effects of the natural bifunctional plant protein CrataBL. These should be proven in preclinical testing using patient-derived GB cells and in animal studies prior to its clinical application.

## 4. Materials and Methods

### 4.1. Chemicals and Reagents

Sodium chloride, ammonium sulfate, bovine serum albumin ≥96% purity, Dulbecco’s Modified Eagle’s Medium (DMEM 5921), l-glutamine, Na-pyruvate, nonessential amino acids, streptomycin, penicillin, 3-(4,5-dimethylthiazol-2-yl)-2,5-diphenyltetrazolium bromide (MTT), trypsin 0.025%, triton X-100, 8.0 μm pore diameter inserts, toluidine blue, heat-inactivated fetal bovine serum (FBS), collagen I, collagen IV, fibronectin, laminin, and phosphate buffered saline 10 mM, pH 7.4 (PBS 1×) were all purchased from Sigma Aldrich (St. Louis, MO, USA). Gelatin was purchased from Calbiochem^®^. 5-Bromo-2′-Deoxyuridine (BrdU), RNase, propidium iodide, annexin-AlexaFluor488, microBCA, C18 column (Vydac) and coomassie brilliant blue were purchased from ThermoFisher Scientific (Grand Island, NY, USA). CM-cellulose resin and Superdex 75 column were purchased from GE Healthcare.

### 4.2. CrataBL Purification

Plant material was collected in Northeast of Brazil in Recife, and identified by the specialist at *Instituto Agronômico de Pernambuco*. A voucher specimen was deposited at the herbarium of the same *Instituto* (n° 61.415). The inhibitor was purified according to Araujo et al., [7] with some modifications. Briefly, the protein was extracted from *Crataeva tapia* bark powder with 0.15 M NaCl (1:20 *w*/*v*) during 12 h agitation. After filtration, the solution was centrifuged at 4000× *g*, 15 min, 4 °C. The soluble crude extract was submitted to ammonium sulfate fractionation, first 0–30% and last step involving 30–60% (*w*/*v*). Lectin content was resuspended and dialyzed in 10 mM citrate phosphate buffer, pH 5.5, and applied to CM-cellulose chromatography column, previously equilibrated with the same dialysis buffer. Absorbed proteins were evaluated by spectrophotometry analyses (A_280_), being CrataBL eluted in dialysis buffer with 0.5 M NaCl. The eluted fraction was injected into a second chromatography (molecular exclusion) Superdex 75 column coupled to an Äkta Avant System (GE Healthcare). Total protein was determined by Lowry protocol following a standard curve with bovine serum albumin. The homogeneity of CrataBL was evaluated by reverse phase chromatography (HPLC) using a C18 column (Vydac) in a linear gradient of acetonitrile in trifluoroacetic acid and by SDS-polyacrylamide gel electrophoresis. The CrataBL activity was analyzed by phenol-sulfuric acid carbohydrates assay in a microplate, as described by Masuko et al. [41].

### 4.3. Cell Lines and Cocultures

Glioblastoma U87-MG cells and bone marrow mesenchymal stem cells (MSCs) were purchased from ATTC^®^ HTB-14™ and Lonza BioScience Walkersville Inc., respectively. MSCs and U87-MG were cultured in DMEM low glucose medium supplemented with 200 mM l-glutamine, 100 mM Na-pyruvate, 1× nonessential amino acids, 100 μg/mL streptomycin, 100 IU/mL penicillin, and 20% (*v*/*v*) heat-inactivated fetal bovine serum (FBS), whereas U87-MG cells were cultured in 10% (*v*/*v*) FBS. The medium was changed every three days. MSC up to passage ten and U87-MG cells up to passage 70 were used. Experiments were performed in triplicates using the medium with 10% FBS. Monolayer cocultures of MSCs and GB cells (U87) were prepared by mixing the cells in 1:1 ratio (MSC/GB cells) that were seeded into monolayer culture plates with formats, corresponding to each particular experiment. The cells were analyzed after 24 and 48 h of direct coculturing.

### 4.4. Cell Viability Assay

The inhibitory effect of CrataBL on the metabolic activity of mesenchymal stem cells, U87 cells, and direct cocultures was measured by reducing NAD(P)H-dependent cellular oxidoreductase enzymes that reflect the number of viable cells present. These enzymes are capable of reducing the tetrazolium dye MTT 3-(4,5-dimethylthiazol-2-yl)-2,5-diphenyltetrazolium bromide to its insoluble formazan form and was performed in triplicate to confirm. Cells were seeded into a 96-well plate: 2000 MSCs, 5000 U87 cells, and in coculture (2000 MSC together with 5000 U87 cells) in 100 μL of medium/well and incubated at 37 °C and 5% (*v*/*v*) CO_2_ overnight. Different concentrations of CrataBL (5–100 μM) were used to treat cells for 24 h and 48 h. MTT substrate (5 mg/mL), 10 μL, was added to the cells in culture upon treatment, followed by 2 h of incubation at 37 °C. The media was then discarded and addition of 100 μL of DMSO enhanced dissolution of crystal formazan, which allowed absorbance (OD) recording at 540 nm using a microplate spectrophotometer (Spectra max Plus 384, Molecular Devices, CA, USA). Percentage of cell viability was calculated in relation to control: Viability (%) = samples OD/control OD × 100%.

### 4.5. Cell Proliferation

BrdU labeling (5-bromo 2′-deoxyuridine) was used to detect new DNA strand synthesis. MSC (2000 cells/well), U87 (5000 cells/well) or coculture cells (mix of MSC and U87 cells used in monoculture) were seeded into a 96-black well plate and incubated for 24 h at 37 °C and 5% (*v*/*v*) CO_2_. CrataBL was added in concentrations of 5, 25, 50, and 100 μM for 24 h and 48 h. Then, BrdU label solution (10 μM) was added per well and incubated for another 4 h at 37 °C. Solution was removed and cells were fixed and denatured for 30 min at room temperature, by a FixDenat solution provided by ThermoFisher Scientific Company—Cell Proliferation ELISA Chemiluminescence Kit. The level of incorporated BrdU was measured by chemiluminescence using anti-BrdU-peroxidase conjugated antibody, diluted 1:100, followed by 120 min incubation at room temperature. After washing, substrate was added and the plate was stirring for approximately 5 min on a shaker. Spectrophotometer FlexStation Multi-Mode Microplate Reader (Molecular Devices) at 405 nm was used to read the light emission of the samples.

### 4.6. Cell Cycle and Cell Death Assay

Cell cycle was analyzed by seeding 100,000 U87 cells, 40,000 MSCs and the mixed cocultures, into 6-well plates containing medium plus 10% (*v*/*v*) FBS, followed by incubation time (24 h at 37 °C and 5% (*v*/*v*) CO_2_). For cell cycle, cells were maintained in 0.2% (*v*/*v*) of FBS for 24 h prior CrataBL treatment (50 and 100 μM) and changed after treatment to medium with 10% FBS for additional 24 h. For analysis, cells were trypsinized and centrifuged at 3000× *g* for 5 min and fixed in ice cold 70% ethanol. For cell cycle cells were stained with a solution composed of 0.1% (*v*/*v*) of triton X-100, 100 μg/mL of RNase and 10 μg/mL of propidium iodide in PBS 1×, after 2 h of cell fixation in cold ethanol 70%. For cell death, 50 μL binding buffer was added to the pelleted cells, containing 2.5 μg/mL of FITC annexin and 5 μg/mL of PI. Dot plots and histograms were analyzed in BD Accuri C6, collecting at least 30,000 events for cell cycle and 10,000 events for cell death, per each experimental condition (BD, San Jose, CA, USA).

### 4.7. Invasion Assay

Cell invasion assay was evaluated by the Boyden Chamber method [42] using inserts with 8.0 μm pore diameter. Inserts were placed into 24-well plates and coated with Matrigel (1:6 in DMEM free FBS) for 30 min at 37 °C to allow polymerization. Cells (MSC 20,000, U87 50,000 and coculture mix) were plated in 250 μL of FBS free medium, after pre-incubation with CrataBL (5–100 μM) for 15 min. Then 400 μL of DMEM complete medium was added to the lower chamber and the plate was kept at 37 °C, 5% (*v*/*v*) CO_2_ for 24 h. Then inserts were washed with PBS 1× and non-invaded cells were gently removed with a cotton swab from the upper surface of the chamber. Membranes were fixed in cold methanol and stained using 1% (*w*/*v*) of toluidine blue for 30 min or overnight, followed by PBS 1× wash. Invaded cells were counted in at least 10 visual fields, under an inverted microscope (Leica, Camera 3000 G and software Leica Application Suite).

### 4.8. Zymography Activity of Metalloproteases

Zymography was performed by electrophoresis with the gel containing 0.2% (*w*/*v*) of gelatin. Cells were seeded into the 96-well plate, 5000 U87 cells/well, 2000 MSC/well and mixed cells for coculture/well and incubated at 37 °C and 5% (*v*/*v*) CO_2_. Upon 24 h, the medium was collected and quantified by MicroBCA assay. 100 µg of total protein was loaded per each lane onto a 7.5% SDS-polyacrylamide separating gels containing gelatin and 5% stacking gel. Gels were run using a BioRad PowerPac apparatus at 100 V, for about 90 min. Then they were washed for 20 min in 2.5% Triton X-100 and incubated in metalloproteases activation buffer (50 mM Tris/HCl, pH 8. 0, 5 mM CaCl_2_, 2 μM ZnCl_2_) or alternatively, in metalloprotease blocking activity buffer (0.5 mM EDTA plus 0.5 mM phenanthroline monohydrate for 16 h at 37 °C. After incubation, the gels were stained with Coomassie solution (40% methanol, 10% acetic acid and 0.1% (*w*/*v*) Coomassie brilliant blue), distained with 10% acetic acid and 40% methanol, and scanned. Gelatinolytic activity of metalloproteases appears as a translucent band with a blue background. In the images, the color scale was inverted to white/black and the band intensity was analyzed by densitometry method with ImageJ software [43].

### 4.9. Cell Adhesion Assay

To measure the adhesion of the cells to the extracellular matrix, different substrates were used for well coating: collagen I (8 μg/well), collagen IV (4 μg/well), fibronectin (4 μg/well), and laminin (4 μg/well). Cells were plated (in mixed cocultured of 20,000 MSCs and 50,000 U87 cells) into 96-well plate in the presence of 100 μL of CrataBL inhibitor (5–100 μM). They were incubated for 4 h at 37 °C and 5% (*v*/*v*) CO_2_. After incubation 1% (*w*/*v*) BSA was added to the cells and they were left for 1 h at 37 °C, followed by washing in PBS 1×. Adhered cells were fixed with 70% cold methanol (*v*/*v*) for 40 min, washed with PBS 1× and stained with 1% of toluidine blue (*w*/*v*) for 30 min. Upon three additional washes with PBS 1×, 1 μL of 1% SDS (*w*/*v*) was added to each well containing 100 μL of PBS 1× for 30 min at 37 °C to solubilize the cells. Absorbance was recorded at 540 nm by microplate reader spectrophotometer (Spectra Max Plus 384, Molecular Devices, Atascadero, CA, USA) [44].

### 4.10. Cytokine Measurement

Cytokine profile of the media was determined in Luminex MAP, using Milliplex MAP Human Cytokine/Chemokine Magnetic Bead Panel (Merck, Kenilworth, NJ, USA) containing G-CSF, GM-CSF, IL-6, IL-8, MCP-1, and VEGF [45]. Cells were seeded into 6-well plate (20,000 MSCs, 50,000 U87 cells, and a mix of cells for coculture) and incubated at 37 °C and 5% (*v*/*v*) CO_2_. At 24 h, CrataBL (50 and 100 μM) inhibitor was added to the cells and the medium was collected after 24 h of treatment. MicroBCA assay was used to determine protein concentrations in media samples against a standard FBS curve, and the samples with 40 μg of the total protein were utilized to perform cytokine analyzes. Medium containing FBS 10% was used as a blank in the measurements.

### 4.11. Nitric Oxide Cell Release

Nitric oxide (NO) release was measured using indirect conversion of nitric oxide to nitrite by a chemiluminescence reaction, in Nitric Oxide Analyzer (NOA^TM^ 208i–Sievers). The NO analyzer was calibrated using a standard sodium nitrite curve ranging from 0.5 μM to 100 μM. Cells were plated into 96-well plate, 5000 cells for U87, 2000 cells for MSCs, and mixed cells for coculture, and incubated for 24 h at 37 °C and 5% (*v*/*v*) CO_2_. Cells were treated with CrataBL (50 and 100 μM) for an additional 24 h and the medium was collected for NO measurement. The protein content of the medium was quantified by MicroBCA assay. The samples containing 100 μg of total protein was evaluated. The medium containing 10% FBS, lacking exposure to the cells, was used as a control.

### 4.12. Statistical Analyses

All experiments were performed in triplicate and independently repeated at least three times. The statistical analyses were expressed as the means ± standard deviation (SD) and analyzed using GraphPad Prisma Software. Comparisons among the variables, measured in defined experimental groups were conducted using one-way ANOVA, followed by Tukey´s test. Statistical significance was defined as * *p* < 0.05, ** *p* < 0.005, and *** *p* < 0.0005.

## References

[1] Mejía E.G., Prisecaru V.I. (2005). Lectins as Bioactive Plant Proteins: A Potential in Cancer Treatment. Crit. Rev. Food Sci. Nutr..

[2] Nunes N.N., Ferreira R.S., Silva-Lucca R.A., de Sá L.F., de Oliveira A.E., Correia M.T., Paiva P.M., Wlodawer A., Oliva M.L. (2015). Potential of the Lectin/Inhibitor Isolated from *Crataeva tapia* Bark (CrataBL) for Controlling *Callosobruchus maculatus* Larva Development. J. Agric. Food Chem..

[3] Lagarda-Diaz I., Guzman-Partida A.M., Vazquez-Moreno L. (2017). Legume Lectins: Proteins with Diverse Applications. Int. J. Mol. Sci..

[4] Zhang F., Walcott B., Zhou D., Gustchina A., Lasanajak Y., Smith D.F., Ferreira R.S., Correia M.T.S., Paiva P.M.G., Bovin N.V. (2013). Structural Studies on the Interaction of *Crataeva tapia* Bark Protein with Heparin and other Glycosaminoglycans. Biochemistry.

[5] Macedo M.L., Oliveira C.F., Oliveira C.T. (2015). Insecticidal Activity of Plant Lectins and Potential Application in Crop Protection. Molecules.

[6] Hashim O.H., Jayapalan J.J., Lee C.S. (2017). Lectins: An effective tool for screening of potential cancer biomarkers. Peer J..

[7] Araújo R.M., Ferreira R.S., Napoleão T.H., Carneiro-da-Cunha M., Coelho L.C., Correia M.T., Oliva M.L., Paiva P.M. (2012). *Crataeva tapia* bark lectin is an affinity adsorbent and insecticidal agent. Plant. Sci..

[8] Ferreira R.S., Zhou D., Ferreira J.G., Silva M.C., Silva-Lucca R.A., Mentele R., Paredes-Gamero E.J., Bertolin T.C., dos Santos Correia M.T., Paiva P.M. (2013). Crystal Structure of *Crataeva tapia* Bark Protein (CrataBL) and Its Effect in Human Prostate Cancer Cell Lines. PLoS ONE.

[9] Salu B.R., Ferreira R.S., Brito M.V., Ottaiano T.F., Cruz J.W., Silva M.C., Correia M.T., Paiva P.M., Maffei F.H., Oliva M.L. (2014). CrataBL, a lectin and Factor Xa inhibitor, plays a role in blood coagulation and impairs thrombus formation. Biol. Chem..

[10] Da Rocha A.A., Araújo T.F., da Fonseca C.S., da Mota D.L., de Medeiros P.L., Paiva P.M., Coelho L.C., Correia M.T., Lima V.L. (2013). Lectin from *Crataeva tapia* Bark Improves Tissue Damages and Plasma Hyperglycemia in Alloxan-Induced Diabetic Mice. Evid. Based Complement. Alternat. Med..

[11] Sottoriva A., Verhoeff J.J., Borovski T., McWeeney S.K., Naumov L., Medema J.P., Sloot P.M., Vermeulen L. (2010). Cancer stem cell tumor model reveals invasive morphology and increased phenotypical heterogeneity. Cancer Res..

[12] Bao S., Wu Q., McLendon R.E., Hao Y., Shi Q., Hjelmeland A.B., Dewhirst M.W., Bigner D.D., Rich J.N. (2006). Glioma stem cells promote radioresistance by preferential activation of the DNA damage response. Nature.

[13] Kucerova L., Matuskova M., Hlubinova K., Altanerova V., Altaner C. (2010). Tumor cell behaviour modulation by mesenchymal stromal cells. Mol. Cancer.

[14] Ullah I., Subbarao R.B., Rho G.J. (2015). Human mesenchymal stem cells—Current trends and future prospective. Biosci. Rep..

[15] Motaln H., Turnšek T.L. (2015). Cytokines play a key role in communication between mesenchymal stem cells and brain cancer cells. Protein Peptide Lett..

[16] Breznik B., Motaln H., Vittori M., Rotter A., Turnšek T.L. (2017). Mesenchymal stem cells differentially affect the invasion of distinct glioblastoma cell lines. Oncotarget.

[17] Kang S.-G., Figueroa J., Gao F., Marini F.C., Hossain A., Sulman E., Gumin J. (2015). Mesenchymal Stem Cells Isolated From Human Gliomas Increase Proliferation and Maintain Stemness of Glioma Stem Cells Through the IL 6/gp130/STAT3 Pathway. Stem Cells.

[18] Oliveira M.N., Pillat M.M., Motaln H., Ulrich H., Lah T.T. (2018). Kinin-B1 Receptor Stimulation Promotes Invasion and is Involved in Cell-Cell Interaction of Co-Cultured Glioblastoma and Mesenchymal Stem Cells. Sci. Rep..

[19] Rasulov M.F., Vasilchenkov A.V., Onishchenko N.A., Krasheninnikov M.E., Kravchenko V.I., Gorshenin T.L., Pidtsan R.E., Potapov I.V. (2005). First experience of the use bone marrow mesenchymal stem cells for the treatment of a patient with deep skin burns. Bull. Exp. Biol. Med..

[20] Chamberlain G., Fox J., Ashton B., Middleton J. (2007). Concise review: Mesenchymal Stem Cells: Their Phenotype, Differentiation Capacity Immunological Features, and Potential for Homing. Stem Cells.

[21] Tajnšek U., Motaln H., Levic N., Rotter A., Lah T.T., Resende R.R., Ulrich H. (2013). Trends in Stem Cell Proliferation and Cancer Research.

[22] Kim S.M., Woo J.S., Jeong C.H., Ryu C.H., Lim J.Y., Jeun S.S. (2012). Effective combination therapy for malignant glioma with TRAIL-secreting mesenchymal stem cells and lipoxygenase inhibitor MK886. Cancer Res..

[23] Gialeli C., Theocharis A.D., Karamanos N.K. (2011). Roles of matrix metalloproteinases in cancer progression and their pharmacological targeting. FEBS J..

[24] Mammoto T., Jiang A., Jiang E., Panigrahy D., Kieran M.W., Mammoto A. (2013). Role of Collagen Matrix in Tumor Angiogenesis and Glioblastoma Multiforme Progression. Am. J. Pathol..

[25] Anestakis D., Petanidis S., Kalyvas S., Nday C.M., Tsave O., Kioseoglou E., Salifoglou A. (2015). Mechanisms and Αpplications of Ιnterleukins in Cancer Immunotherapy. Int. J. Mol. Sci..

[26] Schichor C., Albrecht V., Korte B., Buchner A., Riesenberg R., Mysliwietz J., Paron I., Motaln H., Turnsek T.L., Jürchott K. (2012). Mesenchymal stem cells and glioma cells form a structural as well as a functional syncytium in vitro. Exp. Neurol..

[27] Bartosh T.J., Ullah M., Zeitouni S., Beaver J., Prockop D.J. (2018). Cancer cells enter dormancy after cannibalizing mesenchymal stem/stromal cells (MSCs). Proc. Natl. Acad. Sci. USA.

[28] Melzer C., Yang Y., Hass R. (2016). Interaction of MSC with tumor cells. J. Cell Commun. Signal..

[29] Barcellos-de-Souza P., Gori V., Bambi F., Chiarugi P. (2013). Tumor microenvironment: Bone marrow-mesenchymal stem cells as key players. Biochim. Biophys. Acta.

[30] Bonturi C.R., Motaln H., Silva M.C.C., Salu B.R., Brito M.V., Costa L.A.L., Torquato H.F.V., Nunes N.N.S., Paredes-Gamero E.J., Turnsek T.L. (2018). Could a plant derived protein potentiate the anticancer effects of a stem cell in brain cancer?. Oncotarget.

[31] Kessenbrock K., Plaks V., Werb Z. (2010). Matrix Metalloproteinases: Regulators of the Tumor Microenvironment. Cell.

[32] Motaln H., Gruden K., Hren M., Schichor C., Primon M., Rotter A., Lah T.T. (2012). Human mesenchymal stem cells exploit the immune response mediating chemokines to impact the phenotype of glioblastoma. Cell Transplant..

[33] Albulescu R., Codrici E., Popescu I.D., Mihai S., Necula L.G., Petrescu D., Teodoru M., Tanase C.P. (2013). Cytokine Patterns in Brain Tumour Progression. Mediators Inflamm..

[34] Dinarello C.A. (2000). Proinflammatory cytokines. Chest.

[35] Wcisło-Dziadecka D., Zbiciak M., Brzezińska-Wcisło L., Mazurek U. (2016). Anti-cytokine therapy for psoriasis-not only TNF-α blockers. Overview of reports on the effectiveness of therapy with IL-12/IL-23 and T and B lymphocyte inhibitors. Postepy. Hig. Med. Dosw..

[36] Lee H., Hong I. (2017). Double-edged sword of mesenchymal stem cells: Cancer-promoting versus therapeutic potential. Cancer Sci..

[37] Schwentker A., Vodovotz Y., Weller R., Billiar T.R. (2002). Nitric oxide and wound repair: Role of cytokines?. Nitric Oxide.

[38] Altieri R., Fontanella M., Agnoletti A., Altieri R., Fontanella M., Agnoletti A., Panciani P.P., Spena G., Crobeddu E., Pilloni G. (2015). Role of Nitric Oxide in Glioblastoma Therapy: Another Step to Resolve the Terrible Puzzle?. J. Trans. Med..

[39] Koç O.N., Gerson S.L., Cooper B.W., Dyhouse S.M., Haynesworth S.E., Caplan A.I., Lazarus H.M. (2000). Rapid hematopoietic recovery after coinfusion of autologous-blood stem cells and culture-expanded marrow mesenchymal stem cells in advanced breast cancer patients receiving high-dose chemotherapy. J. Clin. Oncol..

[40] Shah K. (2012). Mesenchymal stem cells engineered for cancer therapy. Adv. Drug Deliv. Rev..

[41] Masuko T., Minami A., Iwasaki N., Majima T., Nishimura S., Lee Y.C. (2005). Carbohydrate analysis by a phenol-sulfuric acid method in microplate format. Anal. Biochem..

[42] Falasca M., Raimondi C., Maffucci T. (2011). Boyden chamber. Methods Mol. Biol..

[43] Lisboa R.A., Andrade M.V., Cunha-Melo J.R. (2013). Zimography is an effective method for detection of matrix metalloproteinase 2 (MMP-2) activity in cultured human fibroblasts. Acta Cir. Bras..

[44] Weitz-Schmidt G., Chreng S. (2012). Cell adhesion assays. Methods Mol. Biol..

[45] Leng S.X., McElhaney J.E., Walston J.D., Xie D., Fedarko N.S., Kuchel G.A. (2008). Elisa and Multiplex Technologies for Cytokine Measurement in Inflammation and Aging Research. J. Gerontol. A Biol. Sci. Med. Sci..

